# MiRNome analysis identifying miR‐205 and miR‐449a as biomarkers of disease progression in intestinal‐type sinonasal adenocarcinoma

**DOI:** 10.1002/hed.26894

**Published:** 2021-10-14

**Authors:** Massimo Re, Marco Tomasetti, Federica Monaco, Monica Amati, Corrado Rubini, Giacomo Sollini, Arisa Bajraktari, Federico Maria Gioacchini, Lory Santarelli, Ernesto Pasquini

**Affiliations:** ^1^ Department of Clinical and Molecular Sciences Polytechnic University of Marche Ancona Italy; ^2^ Department of Biomedical Sciences and Public Health, Anatomy Pathology and Histopathology Section Polytechnic University of Marche Ancona Italy; ^3^ ENT Division “Bellaria Hospital” AUSL Bologna Italy

**Keywords:** biomarkers, ethmoid sinus, intestinal‐type sinonasal adenocarcinoma (ITAC), miRNAs, miRNome

## Abstract

**Background:**

Patients with intestinal‐type sinonasal adenocarcinoma (ITAC) have an unfavorable prognosis, and new diagnostic and therapeutic approaches are needed to improve clinical management.

**Methods:**

Next‐generation sequencing‐based miRNome analysis was performed on 43 ITAC patients who underwent surgical resection, and microRNA (miRNA) data were obtained from 35 cases. Four miRNAs were identified, and their expression levels were detected by reverse‐transcription quantitative polymerase chain reaction and related to the relevant patient outcome. Overall survival and disease‐free survival rates were evaluated through the Kaplan–Meier method and log‐rank test, and multivariate analysis was performed by means of Cox proportional hazard analysis.

**Results:**

High levels of miR‐205 and miR‐34c/miR‐449 cluster expression were associated with an increased recurrence risk and, therefore, a worse prognosis. Multivariate analysis confirmed that miR‐205 and miR‐449 were significant prognostic predictors.

**Conclusions:**

A high expression of miR‐205 and miR‐449 is independent predictors of poor survival for ITAC patients.

## 1 INTRODUCTION

Malignant tumors of the nasal cavity and paranasal sinuses account for 0.2% of all human primary malignant neoplasms, with an incidence of 0.1–1.4 new cases/year/100 000 inhabitants.^1^, ^2^

Adenocarcinomas account for 10%–20% of all primary malignant neoplasms of the sinonasal tract.^3^ Many of these have a salivary gland origin, in which others have histologic patterns resembling those of colon adenocarcinomas. The latter type of sinonasal adenocarcinoma was named intestinal‐type sinonasal adenocarcinoma (ITAC) and is responsible for less than 4% of the total malignancies in this region.^4^ ITACs of the nasal cavity and paranasal sinuses can occur sporadically or are associated with occupational exposure to hardwood and leather dust.^5^ Exposure to wood and leather dust increases the risk of adenocarcinoma by 500‐fold.^6^ Findings from several studies have suggested clinical differences between ITAC arising in individuals with occupational dust exposure and ITAC arising sporadically. In fact, tumors related to occupational exposure affect men in 85%–90% of cases, showing a strong tendency to arise in the ethmoid sinuses.^5^

ITAC seems to be preceded by intestinal metaplasia of the respiratory mucosa, induced by hardwood dust, leather dust, and other unknown agents, accompanied by the switch to an intestinal phenotype.^7^ ITACs are aggressive tumors, characterized by frequent local recurrence, low incidence of distant metastases, with a 40%–60% 5‐year survival rate and local recurrence often occurring within 2 years of follow‐up, being the main contributor to an approximately 53% overall mortality rate.^8^ Histopathological grading and TNM classification appear to be significant prognostic indicators.^9^

Complete surgical resection with negative margins by means of either open or endoscopic approach is the preferred treatment. Postoperative radiotherapy is also usually recommended in an effort to mitigate local recurrence. Multimodality treatments, including chemotherapy, are also used depending on the tumor subtype and stage as well as the experience of individual centers.^10^, ^11^

Although some of the classic prognostic factors are still extremely useful to predict the clinical behavior of ITACs, it is not clear yet why some patients with ITACs have a better prognosis than others with the same type of lesion in terms of histological characteristics and clinical stage.

Conventional clinical pathological parameters do not accurately reflect the clinical outcome of patients with this tumor. Therefore, there is an urgent need for new prognostic factors, which can predict the outcome in patients affected by ITACs. The molecular events involved in the pathogenesis of ITAC^12^, ^13^, ^14^, ^15^, ^16^ are not fully known despite the increasing information about the molecular mechanisms involved in the pathogenesis of head and neck squamous cell carcinomas (HNSCC).^17^, ^18^, ^19^, ^20^, ^21^ Overall, few recurrent gene mutations have been noted in sinonasal adenocarcinoma. The most frequently demonstrated is KRAS gene mutation, which has been detected in 12%–43% of cases, with a potential link to wood‐dust‐associated carcinogenesis.^22^ As well, TP53 mutations and p53 protein overexpression have been found in 18%–44% of mostly occupational ITACs.^15^ Sánchez‐Fernández et al. with the aim to identify actionable gene mutations that can guide new personalized target‐specific therapies in ITAC patients analyzed 27 ITAC germline DNA samples by next‐generation sequencing (NGS), and potentially actionable somatic mutations were found in 20 of 27 cases, eight of which being biomarkers of FDA‐approved targeted therapies.^23^

To compare genomic changes in ITAC and colorectal adenocarcinoma (CRC), a DNA sequencing was performed, and the authors identified several genetic similarities between ITAC and CRC.^24^

Interestingly, Lopez‐Hernandez et al. performed the genetic analysis of 96 ITACs by microarray comparative genomic hybridization and immunohistochemistry, correlated with previously obtained mutation, methylation, and protein expression data, and with pathological characteristics and clinical outcome. This study identified genetic subgroups with distinct clinical outcomes.^14^

Evidence on ITACs genetic findings remains mixed, and probably, the key to a better understanding of the genetic and epigenetic landscape of these tumors lies in a more accurate definition of their genomic and epigenomic profile with improved technologies. Recently, regulatory epigenetic mechanisms were intensively investigated in many fields, and several studies showed that epigenetic alterations play a critical role in HNSCC carcinogenesis.^25^ MicroRNAs (miRNAs), a class of small non‐protein‐coding RNA molecules that control the expression of target genes, are now recognized as crucial components of the epigenome, orchestrating events ranging from organogenesis to immunity, and they are known to be key to the development of many diseases, including cancer.^26^ They regulate gene expression at a post‐transcriptional level by binding to partially complementary sites in the 3'‐untranslated regions (UTRs) of their messenger RNA targets. MiRNAs function as gene expression repressors at the post‐transcriptional level, affecting the translation or causing the degradation of the mRNA targets.^27^ Apparently, miRNAs may act as tumor suppressors or oncogenes by targeting oncogenes or tumor suppressor genes, respectively.^27^ Interestingly, the overall miRNA expression profile in normal tissues is separate from the one in tumor tissues.^28^ MiRNA regulation is jeopardized in cancer, and a number of studies suggested that this dysregulation may be associated with various tumor characteristics and prognosis in a variety of tumor types.^29^, ^30^, ^31^ Though aberrations in miRNA expression in primary HNSCC tumors were recently defined in several reports^32^, ^33^ little is known about how these differences are linked with clinical features and disease outcome.

In this study, the miRNA expression profile of ITACs was evaluated by NGS miRNome analysis, and specific miRNAs involved in the pathology were identified. The selected miRNAs were then detected in a well‐characterized and homogeneous cohort of patients with ethmoidal ITACs, and their prognostic value was predicted.

To the best of our knowledge, there are no previous reports on miRNA expression profile in ITACs.

## 2 MATERIALS AND METHODS

### 2.1 Patient and specimen selection

In this retrospective and prospective multicenter cohort study, data from patients with primary ITACs consecutively treated by primary surgery at the Department of Otorhinolaryngology of the Regional Hospital of the Polytechnic University of Marche, Ancona, Italy, and at ENT Division “Bellaria Hospital” – AUSL Bologna, Italy (both tertiary rhinology referral centers), were retrieved between 2011 and 2017. The medical charts of all patients were reviewed, and the following information was collected: age at diagnosis, sex, occupational history, site of tumor, stage, histological findings, surgery, adjuvant treatment, disease‐free survival (DFS), overall survival (OS), and follow‐up period.

Inclusion criteria were: primary surgical treatment with complete surgical excision of the tumor by means of endoscopic resection with or without trans‐nasal craniectomy (ERTC) or by cranioendoscopic resection, complete clinical data, a minimum of three follow‐up years for patients without recurrence, the availability of surgical and formalin‐fixed paraffin‐embedded (FFPE) tissue samples, and the availability of paired tumor tissues and adjacent normal tissue samples.

Patients with previous or synchronous second malignancies, previous radiation therapy or chemotherapy, or who had died of postoperative complications were excluded from the study.

### 2.2 Ethical approval and consent to participate

At the time of surgery, patients were asked to sign a written informed consent to process their personal anonymized data “for scientific purposes” only. The study was conducted according to the Helsinki Declaration, and the samples were processed after approval of the Ethical Committee of the Marche Regional Hospital, Ancona, Italy, Rec. no. 501 of 29 November, 2011.

### 2.3 Patient cohort and workup

Clinical data were collected prospectively from patients, and then updated retrospectively after the follow‐up review. Overall, 43 patients met the inclusion criteria. All patients had undergone complete clinical examination and were staged by multiplanar CT and contrast enhanced MRI (or contrast‐enhanced CT whenever an MRI could not be obtained), and PET/CT in advanced‐stage lesions. After imaging evaluation, a biopsy of the lesion under local anesthesia was obtained.

Identification of the anatomical site of the tumors (T1–T4), nodal involvement (N0–N3), and clinicopathological stage were determined in accordance with the American Joint Committee on Cancer TNM classification of malignant tumors.^34^ Treatment planning was discussed by the local multidisciplinary team of both centers, based on a common management strategy.

### 2.4 Surgery

All patients were treated by endoscopic resection, with or without trans‐nasal craniotomy (ERTC) or by cranium‐endoscopic resection, based on the local extent of disease. Surgical techniques are detailed described elsewhere.^35^, ^36^

Lesions without any critical relationship with the orbit and anterior skull base were considered amenable to endoscopic resection. Contact, with or limited infiltration of the anterior skull base, and focal infiltration of the dura, with or without limited intradural extension, were considered suggestions for ERTC, which included the resection of the anterior skull base between the two laminae papyracea and from the back wall of the frontal sinus back to the planum sphenoidale, the overlying dura, and olfactory bulbs and tracts. Lesions with front or site involvement of the frontal sinus, infiltration of the dura far over the orbital roof, or extensive infiltration of the brain were managed by combining the endoscopic approach with sub‐frontal craniotomy (cranioendoscopic resection). Biopsy samples of the tissue surrounding the area of the suspected tumor attachment were sent as intraoperative pathological specimens to confirm negative margins after resection. Treatment of the neck was only performed in patients who displayed clinically and radiologically positive nodes upon diagnosis. During surgery, paired samples from tumor tissues and adjacent normal tissues were obtained from each patient and immediately preserved in RNAlater solution (Ambion, ThermoFisher, Milan, Italy), frozen at −20°C, and subsequently stored at −80°C.

### 2.5 Histological evaluation

The diagnosis and assessment of the histological findings, according to the WHO (2005) criteria (papillary–colonic–solid–mucinous and mixed subtypes),^37^ were performed on 4–6 μm‐thick paraffin tissue sections stained with conventional hematoxylin and eosin, by a senior pathologist (C.R.).

### 2.6 Adjuvant therapy

Although advanced stage, poor differentiation, and presence of positive surgical margins were the main factors being considered, the suggestion for adjuvant RT was discussed with each patient by the multidisciplinary team, moreover, considering age, comorbidities, previous treatment, and patient's availability for adequate follow‐up especially for low‐stage ITAC.

### 2.7 Follow‐up

All patients were followed as per our institutional protocols, which included an endoscopic evaluation and magnetic resonance imaging (MRI) every 4 months in the first year, every 6 months until the fifth year, and yearly thereafter.

### 2.8 MiRNome analysis

The first step of the study involved identifying deregulated miRNAs in malignant neoplastic tissue in relation to the adjacent non‐malignant counterpart of the nasal cavity. For this purpose, all miRNAs (miRNome) were analyzed by “NGS” technology. MiRNome analysis was performed on a subgroup of ITAC patients (*n* = 5). Total RNA was extracted from the paired biopsy samples (tumor and adjacent normal tissue) of the selected patients using a commercial RNeasy Mini Kit (Qjagen, Hilden, Germany). After analyzing the purity and integrity of total RNA using the Qubit Flex Fluorometer (ThermoFisher, Milan, Italy), samples were sequenced with NGS technology.

NGS analysis was carried out at the Functional Genomic Lab Department of Biotechnology, Verona University, Italy. The libraries were prepared using the TruSeq RNA Sample Prep Kit v2 (Illumina Inc., San Diego, CA, USA) in accordance with Manufacturer's guidelines, and subsequently sequenced by Illumina NextSeq500 (Illumina Inc., San Diego, CA, USA) “sequencer” that generated ~22 million coupled‐end readings at 75 bp per sample. After quality control with FastQC (version 0.11.3), the data were processed through miRDeep2 (v2.0.0.5) and miRBase (version 21), as previously described.^38^ The DESeq2 package (version 1.8.1) was used to detect the differential miRNA expression in the various conditions. The miRNA levels recovered from the miRDeep2 output were analyzed using R (version 3.2.2). Results were sorted for significantly altered miRNAs by applying a falsehood rate of less than 5%. For isomiR quantification, Isomirage was used to map all readings into an isomiR database derived from a miRBase. The readings were proportionally converted, and the predominant isomiR for miRNA was identified for each condition. The remaining readings from miRDeep2 sequencing were used to predict new miRNAs.

### 2.9 MiRNA detection by quantitative RT‐PCR

Total RNA was extracted from biopsy samples (30 mg) by means of RNeasy (Qiagen, Hilden, Germany) kit as per the manufacturer's instructions. RNA concentration and purity were determined using the Nanodrop 1000 spectrophotometer (ThermoFisher Scientific, Milan, Italy). cDNA was synthesized using a reverse transcription kit TaqMan® Advanced miRNA cDNA Synthesis Kit (item no. A25576; Life Technologies, Grand 23 Island, NY). Quantitative RT‐PCR (qPCR) was performed through the TaqMan Fast Advanced Master Mix by using the following assays (Life Technologies, Grand 23 Island, NY): miR‐205 (ID 477967); miR‐192 (ID 478262); miR‐34c (ID 478051); and miR‐449a (ID 478571). MiR‐99b (ID 478343) was used as a “housekeeping” normalizing gene. PCR assays were performed with Mastercycler EP Realplex (Eppendorf, Milan, Italy) under the following conditions: 50°C for 2 min, 95°C for 20 s, followed by 40 cycles of 95°C for 1 s and 60°C for 20 s, followed by a final step at 4°C. The data were analyzed using the automatic cycle threshold setting (CT) to assign the baseline and the threshold for Ct determination. The samples were analyzed in duplicate, and miRNAs with a CT value of >35 were excluded. The results were expressed as a relative expression (2^−ΔCT^) or fold change (2^−ΔΔCT^).

### 2.10 Statistical analysis

Results are expressed as mean ± SD or as median, quartile, and confidence interval (CI). The categorical variables were reported as fractions or percentages and compared with the chi‐square method. Comparisons between and among groups were performed using Student's *t*‐test two‐tailed (two groups) and by analysis of variance (ANOVA) followed by post hoc Tukey analysis, respectively. Receiver operating characteristic (ROC) curve analysis was used to assess the diagnostic sensitivity and specificity of selected miRNAs (miR‐205, miR‐192, miR‐34c, and miR‐449a), and the area under the ROC curve (AUC) was used as a diagnostic index and for prognostic accuracy. Survival analysis was applied to evaluate the cumulative probability of overall disease survival (OS) and disease‐free survival (DFS). OS was defined as the time interval between the date of surgery and the date of death by disease or other cause, or the date of the latest clinical follow‐up. DFS was defined as the duration between the completion of treatment and the diagnosis of disease recurrence. The cumulative incidence function (CIF) of DFS and OS was estimated by means of the Kaplan–Meier method, and, for each variable, CIFs for different groups were compared using the log‐rank test. Cox proportional hazard model was employed in a multivariate analysis to assess the effect of prognostic factors in DFS and OS. Insignificant prognostic factors were excluded from the model through Wald‐backward elimination. Probability values of <0.05 were considered as significant. All statistical analyses were performed by means of the SPSS statistical package (SPPS Inc., Chicago, IL).

## 3 RESULTS

### 3.1 Patient data

Overall, 43 patients met the inclusion criteria. The patients' main clinicopathological features are summarized in Table 1. All of the 43 patients had a locoregional disease without distant metastases and had undergone radical surgery with tumor‐free resection margins, followed by postoperative radiotherapy and/or chemotherapy in selected cases. There were 40 (93%) males and three (7%) females, with a mean age of 69 ± 13 years. Exposure to wood and leather was documented for 81% of the participants. Tumor localization was ethmoidal in all cases. No patients displayed clinically and radiologically positive nodes upon diagnosis. Pathological TNM classification was distributed as follows: Stage I, pT1N0M0, *n* = 2 (5%); Stage II, pT2N0M0 *n* = 13 (30%); Stage III, pT3N0M0, *n* = 22 (51%); Stage IVa, pT4aN0M0 *n* = 5 (12%); Stage IVb, pT4bN0M0, *n* = 1 (2%).

**TABLE 1:** Overview of the clinical and pathological characteristics of patients with primary ethmoidal ITACs

Characteristics	Values
Age, mean year ± SD	69 ± 13
Sex no. (%)	
Male	40 (93)
Female	3 (7)
Wood/leather exposure	
Yes	35 (81)
No	8 (19)
Type of surgery no. (%)	
ERTC	36 (84)
CR	7 (16)
Adjuvant therapy no. (%)	
None	23 (53)
RT	20 (47)
Subtype	
Mucinous	13 (30)
Colonic	12 (28)
Papillary	16 (37)
Solid	2 (5)
pT classification no. (%)	
T1	2 (5)
T2	13 (30)
T3	22 (51)
T4a	5 (12)
T4b	1 (2)
cN classification no. (%)	
N0	43 (100)
N1	0
N2a	0
N2b	0
N2c	0
N3	0
Relapse no. (%)	
No	24 (56)
Yes	19 (44)
Status	
NED	25 (58)
AWD	0
DOD	18 (42)
DOC	0

Abbreviations: AWD, alive with disease; CR, cranioendoscopic resection; DOC, dead of other causes; DOD, died of disease; ERTC, endonasal endoscopic resection with or without trans‐nasal craniectomy; NED, no evidence of disease; RT, radiotherapy.

### 3.2 Surgery

Endoscopic resection with or without trans‐nasal craniotomy (ERTC) was the most commonly adopted procedure for treatment of the primary lesion (36 patients, 84%). A cranium endoscopic resection was performed in seven patients (16%). No elective treatment of the neck was performed (Table 1).

### 3.3 Histologic findings and postoperative staging

The distribution by histological subtype was as follows: 16 patient (37%) had a papillary subtype, 12 patients (28%) had a colonic subtype, two patients (5%) had a solid subtype, and 13 patients (30%) had a mucinous subtype (Table 1).

### 3.4 Adjuvant therapy

Adjuvant radiotherapy on the primary site with different techniques was delivered to 20 out of 43 patients (47%): conformal 3D‐CRT in 15 (75%) and intensity modulated RT (IMRT) in five patients (25%). The dose ranged from 50 to 66 Gy, in which elective RT on the neck (50 Gy) was added for six patients (Table 1). No chemotherapy was performed.

### 3.5 Follow‐up

Follow‐up information was available with a median of 49.5 months (95% CI: 36.8–68.2 months), and no patients were lost in the follow‐up. During the follow‐up period, 18 patients (42%) developed a local relapse. All patients with local recurrence were treated with a surgical re‐intervention ± RT or RT, and 18 patients (42%) died from the disease, the main cause of death being local recurrence and intracranial invasion. At the time of writing, 25 of 43 patients (58%) remained disease‐free.

### 3.6 MiRNA analysis

#### 3.6.1 Study design

The study included three phases:

Phase I: identification of the miRNAs that are regulated in the tumor tissue compared to their adjacent non‐malignant counterparts.

Phase II: validation of the selected miRNAs.

Phase III: correlation of the selected miRNAs with the patient outcome.

#### 3.6.2 Phase I: MiRNAs identification

**FIGURE 1:**
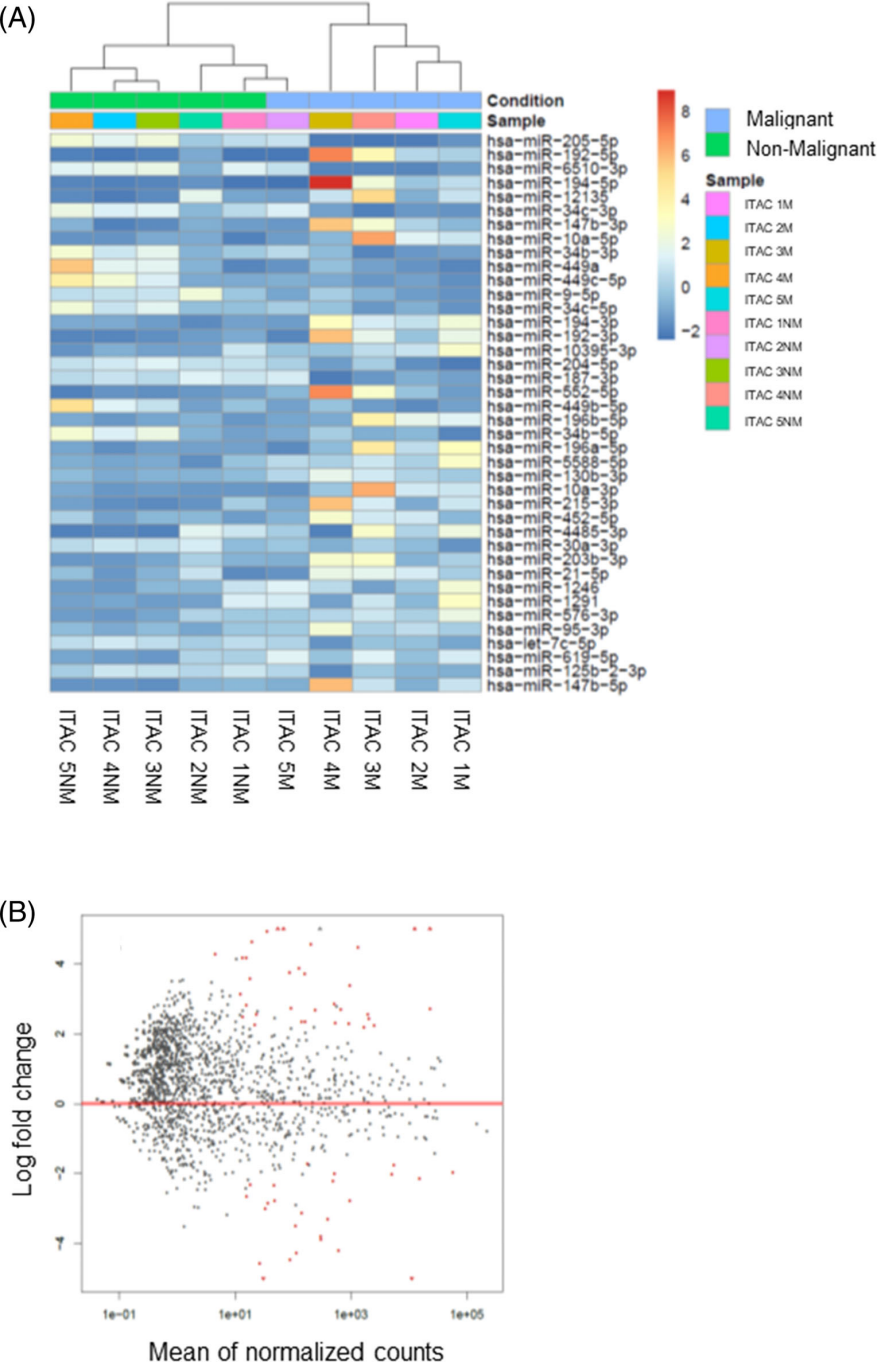
Hierarchical cluster analysis based on miRNA expression (miRNome). (A) Heatmap of significantly deregulated miRNA between malignant (M) and adjacent non‐malignant counterpart (NM). Intensity of color hints at the expression level (red‐orange, high expression; sky‐blue, low expression). (B) Volcano plot between cancer and normal tissue. There were relatively upregulated/downregulated miRNAs with statistical significance (adjusted *p* < 0.05) [Color figure can be viewed at wileyonlinelibrary.com]

**FIGURE 2:**
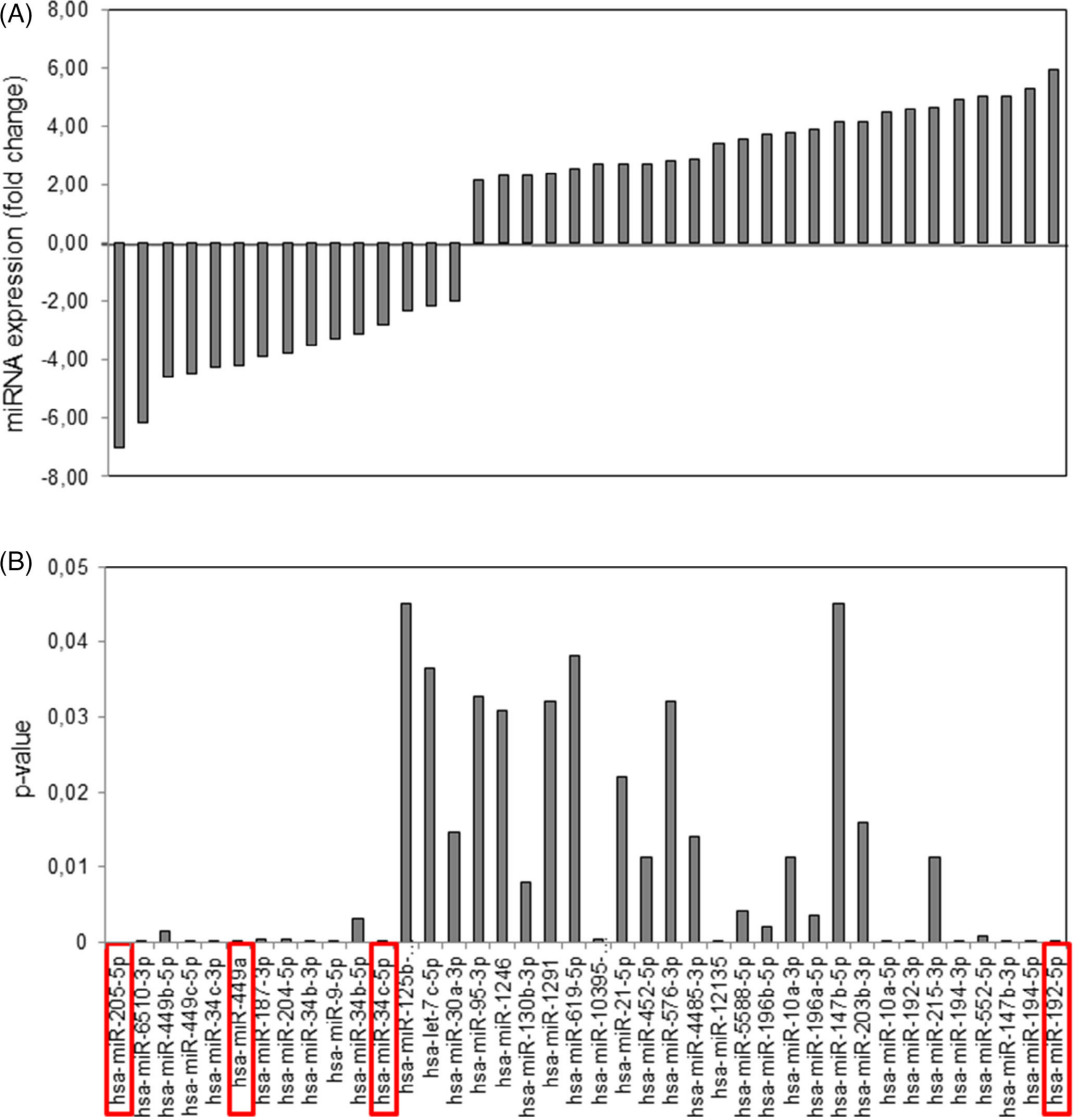
Distribution of deregulated miRNAs in ITAC. Down‐ and upregulated miRNA in malignant tissues compared to non‐malignant tissues according to fold‐change (A) and *p*‐value (B). Differences with *p* < 0.05 were considered statistically significant [Color figure can be viewed at wileyonlinelibrary.com]

MiRNA identification was performed by NGS‐based miRNome analysis on a sub‐group of five ITAC patients (age 70 ± 8 years, four males and one female, two smoking and three no‐smoking, and two mucinous, two colonic, and one papillary subtype). As shown in Figure 1, the heatmap of log‐transformed normalized miRNA expression levels detected 40 deregulated miRNAs in the tumor compared to their corresponding normal counterparts, and most of them were overexpressed in the malignant tissue (62%). Among them, miR‐205 and miR‐192 were the most significantly under‐ and overexpressed miRNAs in tumor tissue, respectively. Also, the miR‐34/miR‐449 cluster, which is highly expressed in multiciliated epithelia,^39^ exhibited the greatest differential expression, which supports their role in tumor (Figure 2).

#### 3.6.3 Phase II: Validation of the selected miRNAs

**FIGURE 3:**
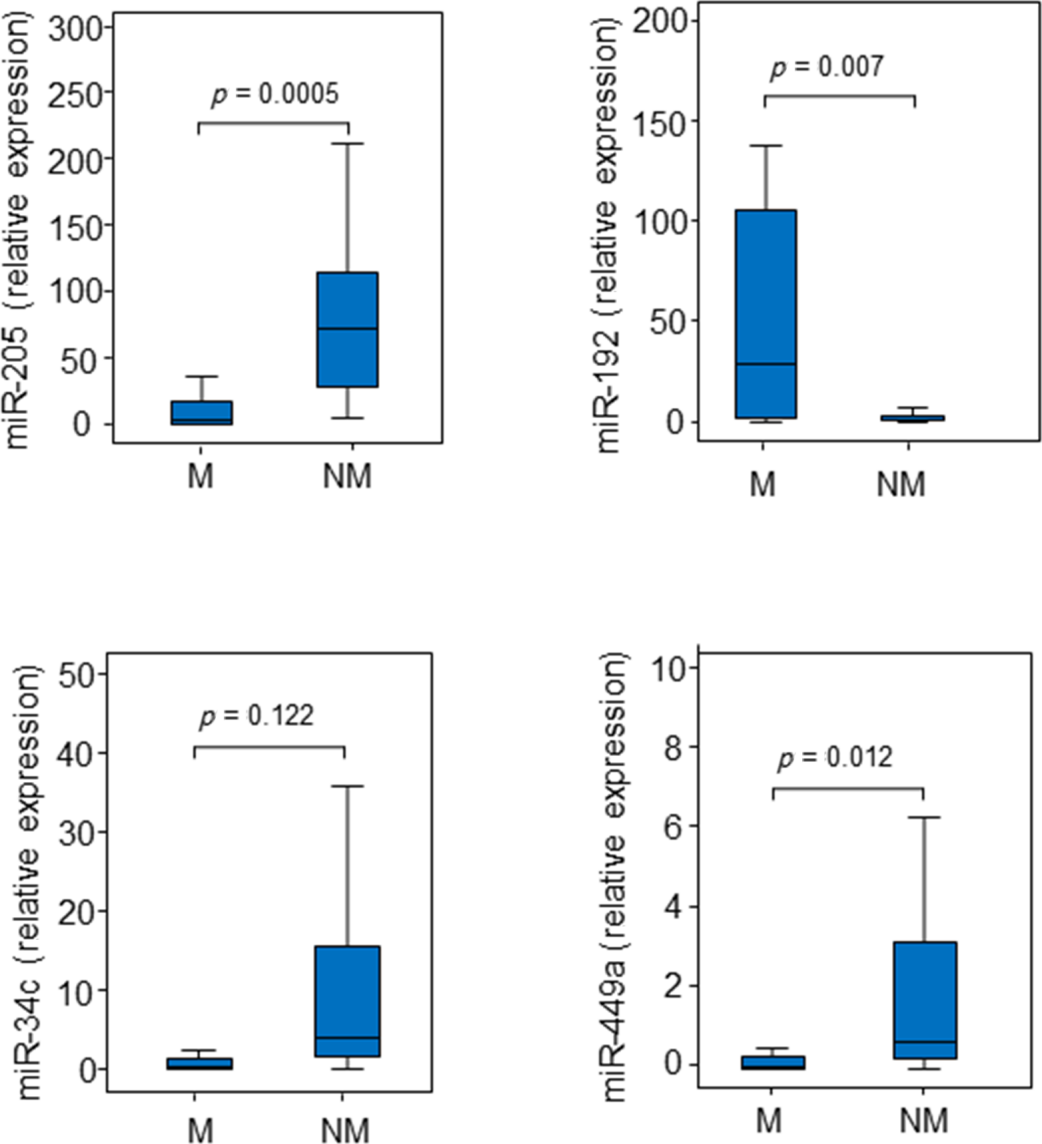
Distribution of selected miRNAs in ITAC. Expression of miR‐205, miR‐192, miR‐34c, and miR‐449a in malignant (M) and non‐malignant (NM) tissues. Comparisons between groups were determined by *t*‐test analysis. Differences with *p* < 0.05 were considered statistically significant [Color figure can be viewed at wileyonlinelibrary.com]

**FIGURE 4:**
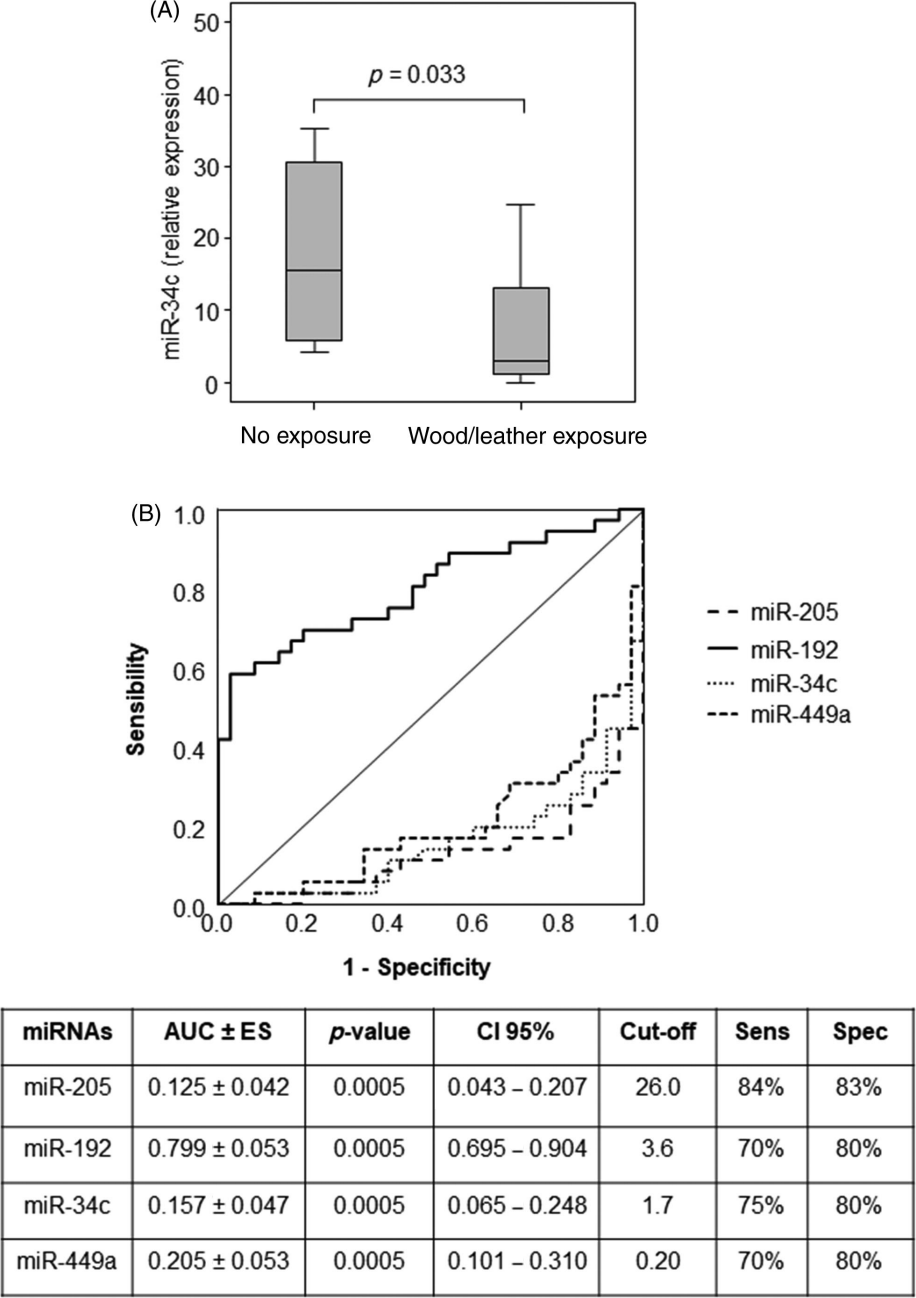
(A) Relationship between occupational exposure and miR‐34c. (B) ROC curves and the area under curve (AUC) with error standard (ES), confidence interval (CI), cut‐offs, sensitivity, and specificity in discriminating ITAC tissues from non‐malignant tissues. Differences with *p* < 0.05 were considered statistically significant

Due to the poor quality of biopsy samples, eight patients were excluded from the analysis, and the identified miRNAs were then validated in 35 ITAC patients by reverse‐transcription quantitative polymerase chain reaction (qRT‐PCR). QRT‐PCR results confirmed the lower expression of miR‐205 and the miR‐34c/miR‐449a cluster and the higher expression of miR‐192 in malignant tissues compared to their non‐malignant counterparts (Figure 3). Among the selected miRNAs, miR‐34c was downexpressed in patients with occupational exposure to wood and leather (Figure 4A). ROC curve analysis revealed that all miRNAs showed high accuracy in differentiating ITAC tissues from non‐tumorous tissues (Figure 4B).

#### 3.6.4 Phase III: Correlation of the selected miRNA with patient outcome

##### Survival analysis

In order to assess their clinical value, the expression levels of the selected 4‐miRNAs were correlated with the outcome of the patients with sinonasal ITAC. DFS and OS were analyzed by means of the Kaplan–Meier curve, and miRNA groups (below and above the median rate) were compared by means of the log‐rank test. Statistically significant variables were entered into a multivariate Cox regression model.

##### Disease‐free survival

**FIGURE 5:**
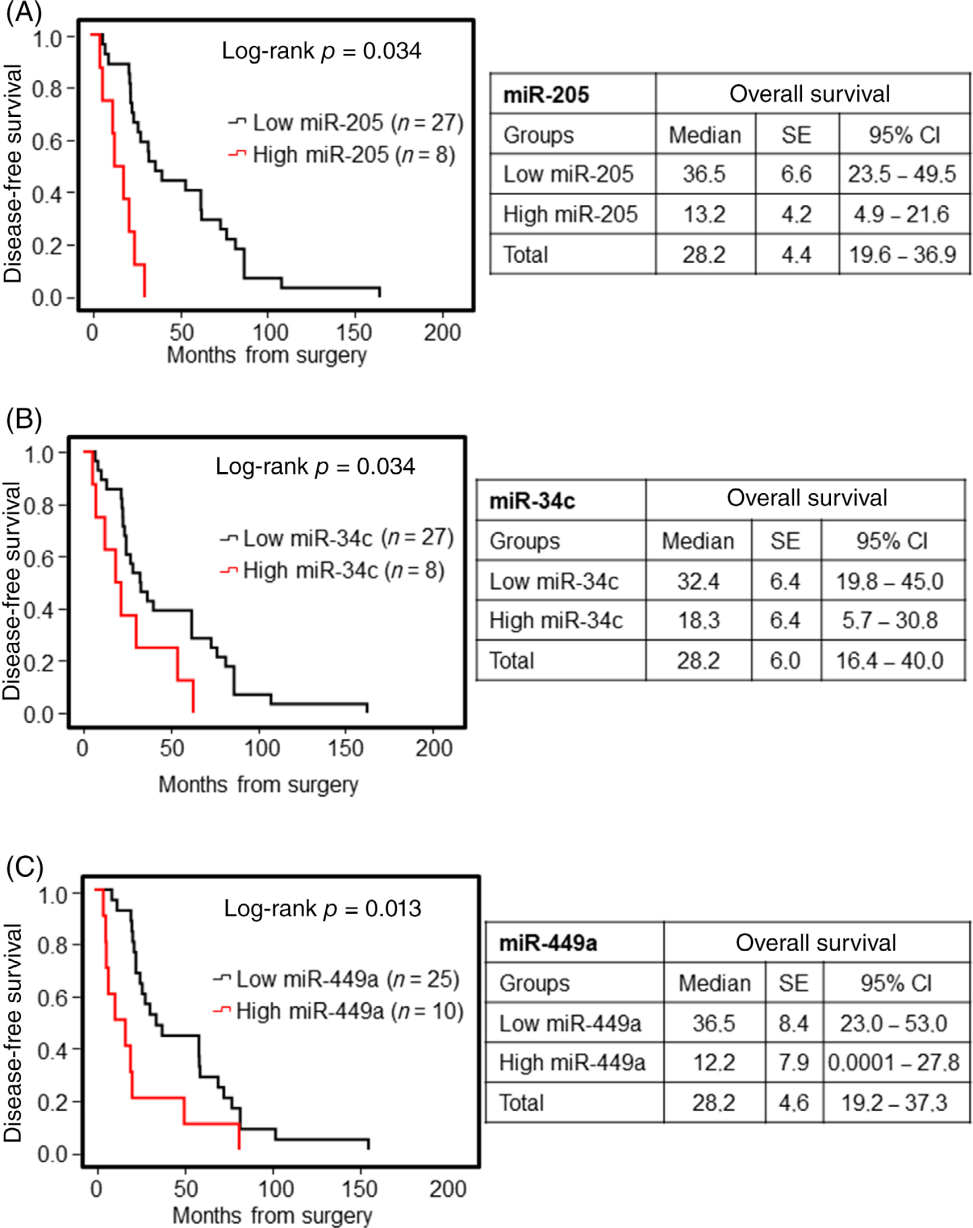
Kaplan–Meier survival curves for ITAC stratified for miRNA expression. Low and high miRNA expressions of miR‐205 (A), miR‐34c (B), and miR‐449a (C) were associated with disease‐free survival (DFS). Comparisons between groups were made using log‐rank test, and two‐sided *p* < 0.05 was considered statistically significant. Median values and a 95% confidential interval (CI) [minimum–maximum] are summarized in the chart on the right [Color figure can be viewed at wileyonlinelibrary.com]

As far as DFS is concerned, miR‐205, miR‐34c, and miR‐449a were significant prognostic predictors for DFS through univariate analysis (Figure 5). Specifically, high expression levels of these miRNAs were associated with a higher recurrence risk and, therefore, worse prognosis.

Multivariate analysis with Cox proportional hazard model confirmed that miR‐205 and miR‐449a were significant prognostic predictors for DFS (Table 2).

**TABLE 2:** Multivariate Cox regression analysis associated with DFS

Variable	HR	95% CI (HR)	*p*‐value
Age	1.00	0.94–1.05	0.86
Sex	‐	‐	‐
Smoking			0.068
Non‐smoker versus smoker	0.23	0.06–0.95	**0.028**
Non‐smoker versus formers	0.15	0.03–0.68	**0.045**
Subtype			0.252
Mucinous versus colonic	0.23	0.05–1.14	0.258
Mucinous versus papillary	0.14	0.03–0.73	0.278
Staging T1 + T2 versus T3 + T4	0.92	0.13–6.63	0.93
miR‐205 low versus high	0.07	0.01–0.41	**0.003**
miR‐192 low versus high	1.86	0.23–15.39	0.56
miR‐34c low versus high	4.78	1.10–20.80	**0.037**
miR‐449a low versus high	0.09	0.02–0.38	**0.001**

*Note*: Regression model with stepwise Wald‐backward adjusted for age, sex, smoking, grading, staging, and miRNAs.

Abbreviations: CI, confidence interval; DFS, disease‐free survival; HR, hazard ratio.

The *p*‐values highlighted in bold are statistically significant.

##### Overall survival

**FIGURE 6:**
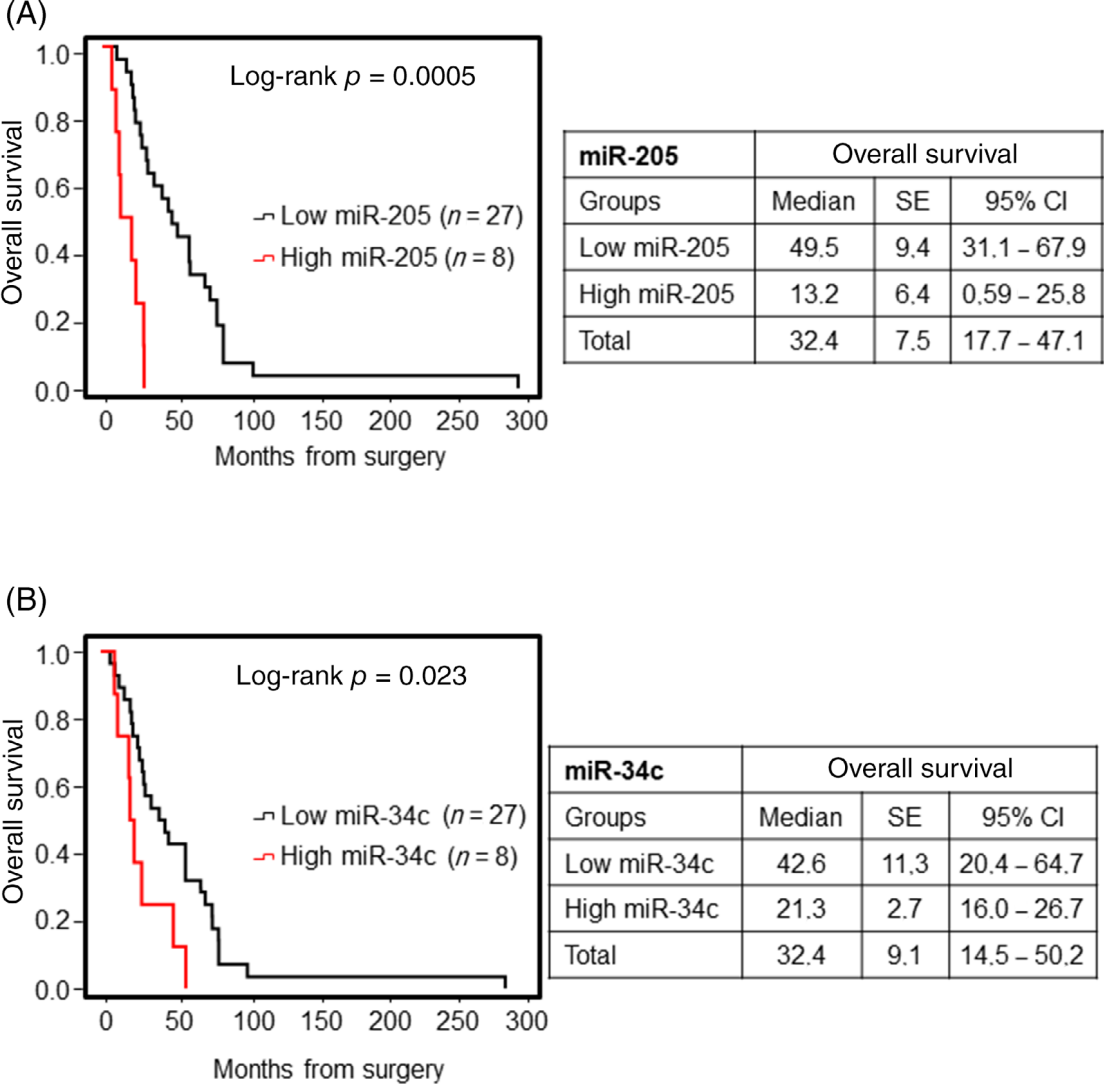
Kaplan–Meier survival curves for ITAC stratified for miRNA expression. Low and high expressions of miR‐205 (A) and miR‐34c (B) were associated with overall survival. Comparisons between groups were made using log‐rank test, and two‐sided *p* < 0.05 was considered statistically significant. Median values and a 95% confidential interval (CI) [minimum–maximum] are summarized in the chart on the right [Color figure can be viewed at wileyonlinelibrary.com]

As far as OS is concerned, miR‐205 and miR‐34c were significant prognostic predictors for OS through univariate analysis (Figure 6); specifically, high expression levels of these miRNAs were associated with worse OS (OS, median 13.2 [95% CI: 0.59–25.8] months vs. 49.5 [95% CI: 31.1–67.9] months, for miR‐205 and OS, median 21.3 [95% CI: 16.0–26.7] months vs. 42.6 [95% CI: 20.4–64.7] months, *p* = 0.023 for miR‐34c). Multivariate analysis with Cox proportional hazard model confirmed that miR‐205 was a significant prognostic predictor for OS (Table 3).

**TABLE 3:** Multivariate Cox regression analysis associated with OS

Variable	HR	95% CI (HR)	*p*‐value
Age	0.98	0.92–1.03	0.39
Sex	‐	‐	‐
Smoking			0.051
Non‐smoker versus smoker	0.16	0.04–0.71	**0.016**
Non‐smoker versus formers	0.27	0.07–1.06	0.061
Subtype			0.423
Mucinous versus colonic	1.91	0.37–9.83	0.442
Mucinous versus papillary	0.51	0.09–02.75	0.430
Staging T1 + T2 versus T3 + T4	0.16	0.03–1.03	0.054
miR‐205 low versus high	0.01	0.001–0.09	**0.0005**
miR‐192 low versus high	1.80	0.30–10.49	0.515
miR‐34c low versus high	4.86	1.04–20.80	**0.044**
miR‐449a low versus high	0.17	0.04–0.65	**0.010**

*Note*: Regression model with stepwise Wald‐backward adjusted for age, sex, smoking, grading, staging, and miRNAs.

Abbreviations: CI, confidence interval; HR, hazard ratio; OS, overall survival.

The *p*‐values statistically significant are highlighted in bold.

## 4 DISCUSSION

ITACs are very aggressive tumors, and, despite the multidisciplinary management with multimodal treatment (surgery and radiotherapy), they are characterized by generally very poor prognosis,^40^, ^41^, ^42^ and, in this context, identification of new diagnostic and prognostic biomarkers is critical to improve the outcome of these patients. Partly due to its low incidence, there is little information about the molecular events involved in ITAC pathogenesis and even fewer studies that can link genetic findings with clinicopathological and follow‐up data.

Many studies have reported significant associations between miRNA profiles and important clinical features concerning head and neck tumors and patient survival.^32^, ^33^, ^43^

At present, due to the rarity of this type of cancer, there are very few studies in literature evaluating of miRNA expression in intestinal‐type adenocarcinomas of the paranasal sinuses.

Recently, downregulated miR‐126 was found in the serum of patients with ITACs, and its re‐expression in sinonasal cancer cells via exosomes promoted anti‐tumor response.^33^

Given these findings, to explore other pathways involved in the molecular pathogenesis of sinonasal ITACs and investigate their potential roles in tumor development, we performed miRNome analysis with NGS technology in patients with ethmoidal ITACs and selected four deregulated miRNAs (miR‐205, miR‐192, miR‐34c, and miR‐449a). In order to analyze their prognostic role, miRNA expression levels were then retrospectively correlated with the clinical pathological characteristics of the tumor itself and patient outcome, so as to evaluate their independent prognostic relevance.

The results of our study showed that miR‐205, miR‐34c, and miR‐449a were downexpressed in cancer tissue compared to their non‐malignant counterparts, and their upregulation in tumor tissue significantly correlated with worse DFS and worse OS. These results suggest that the upregulation of these miRNAs in ITACs may be linked with higher tumor aggressiveness. Overall, miR‐205 and miR‐449 may play a key role in the progression of human ITACs and could be promising markers to predict the occurrence of relapse.

Based on our results, miR‐449a was found to be underexpressed in numerous other cancers, including lung, colorectal, gastric, cervix, and prostate cancer.^44^, ^45^, ^46^, ^47^, ^48^ Conversely, upregulated miR‐449a was found in malignant breast tissue where its high expression was significantly associated with a reduction in DFS and OS.^49^

As for miR‐205, several studies found a high expression of miR‐205 in head and neck squamous cancer cell lines and esophageal squamous cell carcinoma,^50^ as well as in lung squamous cell carcinoma and bladder cancer,^51^ but none of them correlated with patient outcome.

On the other hand, a low miR‐205 expression was found in breast cancer,^52^ prostate,^53^ renal,^54^ and malignant melanoma,^55^ eliciting its tumor suppressive function. A wide range of evidence supports its dual role in cancerogenesis, acting either as a tumor suppressor by inhibiting proliferation and invasion, or as an oncogene by facilitating tumor initiation, proliferation, and resistance to anti‐tumor therapy depending on the specific tumor context and target genes.^54^ In addition, miR‐205 is speculated to potentially target over 2000 genes if all available algorithms are taken into account, and a micro‐network may be established between miR‐205 and its multiple targets.^56^

ITACs arise from intestinal metaplasia of the ciliated respiratory epithelium and mimic the appearance of the mucosa in normal and neoplastic intestine.^57^ Notably, miR‐205 and the miR‐34/miR‐449 cluster are involved in the organization of epithelial tissues. While miR‐205 regulates the structure of the epithelium, the miR‐34 (miR‐34b and miR‐34c)/miR‐449 (miR‐449a, miR‐449b, and miR‐449c) cluster is essential in the development of ciliated epithelia.^56^, ^58^ Altogether, these findings clearly support the essential role of both miR‐205 and miR‐449 in maintaining epithelial biogenesis, and an alteration of these miRNAs may contribute to cancer development.

Interestingly, miR‐449, located in the second intron of CDC20B in chromosome 5q11.2, has some sequence similarities with miR‐34 family, suggesting similar mRNA targets,^59^, ^60^ and is classified as a single miRNA family involved in the proper differentiation of airway epithelium and in the first line of defense of the respiratory tract against toxic agents.^61^

Two model systems (Xenopus embryos and cultured human cells) revealed that miR‐449 is essential for the development of ciliated epithelia, and this appears to depend on miR‐449‐mediated modulation of the Notch signaling pathway.^62^ A double negative feedback loop between Notch and miR‐449 was described: miR‐449 represses Notch activity, which enhances miR‐449 expression in return. Moreover, the impairment of cilia architecture and ciliogenesis was associated with the downregulation of miR‐449 superfamily.^63^

MiRNAs act as extracellular messengers of biological signals coming from the cross‐talk between the tumor and its surrounding microenvironment. Therefore, deregulated miRNAs can be detected in circulation and used as non‐invasive biomarkers for diagnosis and monitoring treatment response of a variety of diseases, including cancer.^64^

However, deregulated miRNAs in tissue specimens were rarely detected in serum samples. This is the case of miR‐126, whose low level was found in the serum of ITAC patients compared to healthy controls, even though it was not differentially expressed in ITAC tissue when compared to its non‐malignant conterpart.^33^

While the differential expression of miRNAs in biopsy is related to tissue features (tissue specificity), the pool of circulating miRNAs is the results of the miRNAs released in the bloodstream by the whole cells of the organism, and often does not mirror the changes observed in tissues. Therefore, considering the high tissue‐specificity of miRNAs, we envision that the miRNA‐profile of biopsy samples could be better used in clinical practice and may help to predict recurrence in ITAC.

The current study has some limits: first of all, although we tried to evaluate a homogeneous patient cohort in terms of stage and treatment, ours is a retrospective (i.e., historical) cohort study, and the patient cohort remains heterogeneous in some critical clinical aspects (TNM different classification, non‐uniform surgical approach, method, and efficacy of complementary protocol treatment, follow‐up). Moreover, the study did not conduct a sensitivity analysis due to the small sample size (*n* = 35). Finally, understanding the connections between miRNAs deregulated in cancer and cellular signaling pathways involved in cancer was hindered by our limited knowledge of miRNA target recognition.

However, considering that one of the most critical clinical elements, which may influence the results being obtained, could be the surgical margin and lymph node status, in our evaluation, no patients had a tumor in or near (<1 mm) the surgical margins or lymph node metastases.

On the whole, despite these limits, the clinical aspects of our highly standardized molecular analysis study of 35 ITACs do not significantly affect the relevance of the obtained results. Even considering that miRNAs can be detected in FFPE tissues, even when the purified RNA is low quality, miR‐449 and miR‐205 could be easily detected from diagnostic biopsies and might potentially help predict the outcome of ITACs in a routine setting.

## 5 CONCLUSIONS

This study provides the first evidence that high expressions of miR‐205 and miR‐449a are independent predictors of poor survival for patients with ITAC. However, in order to confirm the effectiveness of the prognostic miRNAs under study, our findings should necessarily request validation through a broader perspective and multicenter randomized series.

## FUNDING STATEMENT

Open Access Funding provided by Universita Politecnica delle Marche within the CRUI‐CARE Agreement. WOA Institution: Universita Politecnica delle Marche.

## CONFLICT OF INTEREST

The authors declare that they have no conflict of interest.

## AUTHOR CONTRIBUTIONS

Massimo Re, Marco Tomassetti, Monica Amati, and Corrado Rubini: Conception and design. Federica Monaco, Marco Tomasetti, Monica Amati, and Massimo Re: Analysis and interpretation of data. Corrado Rubini, Giacomo Sollini, Arisa Bajraktari, Federico Maria Gioacchini, and Ernesto Pasquini: Patients' recruitment, demographic and clinic data. Marco Tomasetti and Massimo Re: Statistical analysis. Massimo Re, Marco Tomasetti, and Lory Santarelli: Manuscript editing and revising. Ernesto Pasquini and Lory Santarelli: Study and approval of the final version of the manuscript.

## Data Availability

The data that support the findings of the study are available from corresponding authors upon request.
